# London Doctors

**Published:** 1875-10

**Authors:** 


"*London Doctors.*

"Sir William Jenner commenced life as an apothecary in a small back street in London, and for a long time the battle of life fell hardly on him. He worked with rare energy, and after obtaining the M. D. degree, and being elected a Fellow of the College of Physicians, was appointed Physician to University College Hospital, a post which he has held uninterruptedly ever since. His gentle, suave manner soon endeared him to his students and pupils, and aided him in securing a high-class practice. At the present time he is physician to the Queen and to the Prince of Wales.

"Another medical luminary commenced life under still more humble auspices. Sir William Gull, when a boy, was engaged to sweep out the surgery and dispensary of Guy's Hospital, an institution to which he is now the consulting physician. He has the largest fashionable practice of any man in Europe, a result due in a greater degree to his fine impressive presence than to any intrinsic worth he possesses. His warmest admirers cannot say that he ever performed any original work calculated to advance medical science. He has published a few papers on different subjects, the best of which is that on 'Abscess of the Brain,' now incorporated in Reynold's System of Medicine.

"Sir Henry Thompson is said to have been originally a draper's shopman, after which he became for a short time a field preacher or ranter. His natural bias led him to attend a course of medical lectures, and he soon devoted himself heart and soul to the study of medicine and surgery. In 1851 he passed the examination of bachelor of medicine, at the University of London, and two years subsequently was elected a fellow of the College of Surgeons. He was for some years surgeon to the late King of the Belgians, on whom he performed lithotrity fifteen or sixteen times, receiving as a fee the sum of three thousand pounds. He has for a long time been the Professor of Surgery to University College Hospital, an appointment he has relinquished within the past few weeks. Apart from his capabilities as an operator, he is a most accomplished painter, and there is seldom an exhibition at the Royal Academy without a valuable contribution from his facile hand. It will be remembered that he was in constant attendance on the Emperor Napoleon III., and it is generally supposed that his fees in connection with the case amounted to quite a little fortune. He is an earnest and uncompromising teetotaller.—*London letter in American Weekly, June 5, 1875.—Clinic.*"

We publish the above extract for the encouragement, not only of a large number of our American medical students, but American medical colleges, and with the hope that the personal efforts of the one and the policy of the other may be rewarded by a fair average of medical luminaries who have come up from the humble walks of life. In the opinion of some, it matters very little where, or how a student may have been born, or how reared, either to himself, the world or our colleges, or how little he may know when entering upon his course of studies, if, like these bright exemplars, he should, after all, become a great luminary in his profession. If the average success is not very low in America, we ought to have some interesting statistics on this subject, for the reason that some of our colleges, at least, furnishing a number every year, who are in a condition to honor themselves by working up from the ground. While occasional instances of self-made men of distinction in the medical world may have influenced professors in some of our colleges to discard proper preliminary education as a necessity, we regard it hazardous, as well to the profession as to the student, to risk the chances of attaining even professional respectability without due educational preparation. While one may succeed, thousands will fail.

Abandoning jests, we advise all who possibly can to prepare themselves by the best educational opportunities of the country, but to all students, whether educated or not, we offer seriously this advice: Seek the very best medical colleges, such as are provided with professors of high attainments and the means required for complete illustration and demonstration, and we confess that a student, whose duty it may be in the beginning to sweep the building of such an institution, may learn more than if admitted to the highest privileges afforded by some others.

